# Administered circulating microparticles derived from lung cancer patients markedly improved angiogenesis, blood flow and ischemic recovery in rat critical limb ischemia

**DOI:** 10.1186/s12967-015-0381-8

**Published:** 2015-02-15

**Authors:** Jiunn-Jye Sheu, Fan-Yen Lee, Christopher Glenn Wallace, Tzu-Hsien Tsai, Steve Leu, Yung-Lung Chen, Han-Tan Chai, Hung-I Lu, Cheuk-Kwan Sun, Hon-Kan Yip

**Affiliations:** Division of Thoracic and Cardiovascular Surgery, Kaohsiung Chang Gung Memorial Hospital and Chang Gung University College of Medicine, Kaohsiung, Taiwan; Department of Plastic Surgery, University Hospital of South Manchester, Manchester, UK; Division of Cardiology, Department of Internal Medicine, Kaohsiung Chang Gung Memorial Hospital and Chang Gung University College of Medicine, Kaohsiung, Taiwan; Center for Translational Research in Biomedical Sciences, Kaohsiung Chang Gung Memorial Hospital and Chang Gung University College of Medicine, Kaohsiung, Taiwan; Department of Emergency Medicine, E-DA Hospital, I-Shou University, Kaohsiung, Taiwan; Institute of Shock Wave Medicine and Tissue Engineering, Kaohsiung Chang Gung Memorial Hospital and Chang Gung University College of Medicine, Kaohsiung, Taiwan

**Keywords:** Lung cancer, Circulating microparticles, Critical limb ischemia, Angiogenesis, Blood-flow restoration

## Abstract

**Background:**

We hypothesized that lung cancer patient’s circulating microparticles (Lc-MPs) could promote angiogenesis, blood flow in ischemic zone and ischemic recovery in rat critical limb ischemia (CLI).

**Methods:**

To investigate the impact of MP therapy on reversing the setting of CLI, adult-male Sprague–Dawley rats (n=50) equally randomized into sham control (SC) (group 1), SC-Lc-MPs (1.0 x 10^7^ particles) (group 2), CLI (group 3), CLI-Hs-MPs (MPs from healthy-subject) (group 4), and CLI-Lc-MPs (group 5) were sacrificed by post-CLI day-14.

**Results:**

In vitro study showed that Lc-MPs enhanced VEGFR2 expression, angiogenesis, nitric-oxide production, and endothelial cell proliferation (all p<0.005). By days 7 and 14, Laser Doppler showed significantly higher ischemic/normal blood-flow ratio in groups 1 and 2 compared with group 3, and was significantly higher in group 4 and further elevated in group 5 (p<0.0001). Numbers of small vessels and endothelial markers (CD31^+^ and vWF^+^ cells) and protein expressions (eNOS, CD31) exhibited a pattern identical to Lasre Doppler among the five groups (all p<0.001). Pro-angiogenic factors (VEGF, CXCR4, SDF-1α, HGF) at cellular and protein levels showed a significant step-wise increase from groups 1 and 2 to groups 3, 4, and 5 (all p<0.001). Protein expressions of fibrotic (Smad3, TGF-β) and apoptotic (mitochondrial Bax, cleaved caspase 3, and PARP) biomarkers displayed an opposite pattern compared to that of Laser Doppler, whereas the protein expressions of anti-fibrotic (Smad1/5, BMP-2) and anti-apoptotic (Bcl-2) biomarkers showed an identical pattern compared with that of Laser Doppler among groups 1 to 3, and 5 (all p<0.001).

**Conclusion:**

Administration of Lc-MPs augmented angiogenesis and restored blood flow in a rat of CLI.

**Electronic supplementary material:**

The online version of this article (doi:10.1186/s12967-015-0381-8) contains supplementary material, which is available to authorized users.

## Introduction

Lung cancer (LC) is the most common cause of cancer-related mortality worldwide [1,2] with non-small-cell lung cancer (NSCLC) accounting for approximately 85% of all cases. Approximately 70% of NSCLC patients who present with locally advanced or metastatic disease have a poor prognosis, with an expected 5-year survival rate of <5% [3,4]. Despite various combinations of surgery, radiotherapy and chemotherapy [2,5,6], overall survival rates remain poor [7-10]. A better understanding of what is the key factors for locally advanced and metastatic disease states may be very important for improving LC therapeutic outcomes.

Microparticles (MPs) are identified as “small plasma membrane fragments” of cells shed into the circulation by activated and/or apoptotic cells in response to changes such as physiological stimulations (thrombin, endotoxin or shear stress), stress, cellular apoptosis (growth factor deprivation or apoptotic inducers), and/or neoplastic transformation [11-16]. Interestingly, MPs appear to have differential roles in angiogenesis depending on their origin [14-20]. Of note, MPs exhibit pro-angiogenic activity, in that they promote formation of capillary-like structures and pro-angiogenic factor production [14,16,17,19,20]. Accumulating published evidence indicates that numbers of circulating MPs are increased in various type of cancer, including LC [21-23]. We have also recently displayed that circulating levels of MPs are significantly increased in LC patients, and circulating levels of endothelial-derived apoptotic MPs are significantly associated with different LC cell types [24]. These studies raise the possibility that circulating MPs are not only biomarkers but may also directly participate in angiogenesis for promoting nutrient delivery to LC cells, local invasion and metastasis. However, these potentially important propositions remain untested in the literature. To extend our recent work [24], the aims of this study, which included both in vitro and in vivo studies, were to address: (1) whether circulating MPs derived from LC patients promoted angiogenesis, differentiation, nitric oxide (NO) production and vascular endothelial growth factor receptor (VEGFR) expression in culturing human umbilical vein endothelial cells (HUVECs); and (2) whether administration of MPs rescued rat critical limb ischemia (CLI) mainly through angiogenesis and enhanced blood flow in ischemic regions.

## Materials and methods

### Ethics

All animal experimental procedures were approved by the Institute of Animal Care and Use Committee at Kaohsiung Chang Gung Memorial Hospital and performed in accordance with the Guide for the Care and Use of Laboratory Animals (NIH publication No. 85–23, National Academy Press, Washington, DC, USA, revised 1996).

### Rationale for the source and dosage of MPs

Because of the results of our recent study [24] that demonstrated strong correlation between the circulating level of MPs and poor prognostic outcome of patients at advanced stages of NSCLC (i.e., stage IIIb or staged IV of NSCLC upon presentation), MPs were obtained from these patients in the present study.

First of all, the safety and efficacy of different regimens of MP administration were compared in the current study. Second, the amount of MPs that could be obtained from one patient was determined based on our knowledge that usually about 1.0 × 10^7^ MPs could be harvested from10 mL of patient’s blood. Therefore, our pilot study used four animals for verification of dosage of MPs for the current study, including one with CLI only and another three animals receiving 1.0 × 10^6^, 5.0 × 10^6^ and 1.0 × 10^7^ MPs, respectively. By day 14, the results of Laser Doppler showed that the blood flow to the ischemic limb was highest in animals treated with the high-dose regimen (i.e., 1.0 x 10^7^ MPs) without notable complication or tumorigenesis. Therefore, the high-dose regimen (1.0 × 10^7^ MPs) was used in subsequent experiments.

### Animal model of critical limb ischemia, animal grouping, and treatments

The procedure and protocol were based on our previous reports [25,26]. In brief, male Sprague–Dawley (SD) rats in CLI groups were anesthetized by inhalation of 2.0% isoflurane. The rats were placed in a supine position on a warming pad at 37°C with the left hind limbs shaved. Under sterile conditions, the left femoral artery, small arterioles, circumferential femoral artery and veins were exposed and ligated over their proximal and distal portions before removal. To avoid the presence of collateral circulation, the branches were removed together. For Laser Doppler study, 10 rats in each group were utilized and 8 rats in each group were used for cellular-molecular assessment. For animals that served as normal controls with and without receiving MPs, the arteries were only isolated without ligation.

The SD rats (n=40) were equally divided into four groups: group 1 [sham control (SC) + 1.0 ml phosphate buffered saline (BPS) (0.5 ml from penile vein injection and 0.5 ml via intra-muscular injection], group 2 [SC + lung cancer-derived (Lc)-MPs (1.0 × 10^7^ particles) in 1.0 cc BPS given as SC group)], group 3 (CLI only), group 5 (CLI + Lc-MPs, 5.0 × 10^6^ particles in 0.5 ml BPS via penile vein injection and 5.0 × 10^6^ particles in 0.5 ml BPS by intra-muscular injection into ischemic zone) just after CLI induction. The rationale for administration of MPs from both intravenous and intra-muscular injection were to consider that combined circulatory and localized therapy with MPs in a situation of CLI would be offer a great enhancement of angiogenesis effect.

In the present study, another 10 animals (group 4) serves as positive controls that received MPs from healthy subjects (Hs) (i.e., Hs-MPs) after the CLI procedure to verify the potential capacity of enhancing angiogenesis of Hs-MPs in ischemic limbs. Laser Doppler, immunohistochemical (IHC) staining, immunofluorescent (IF) and Western blot studies were used for measuring blood flow, identification of small blood vessel, and quantification of cellular elements of angiogenesis [i.e., CD31^+^, CXCR4^+^, stromal cell-derived factor (SDF)-1α^+^, von Willebrand factor (vWF)^+^ and VEGF^+^ cells], respectively. All animals were sacrificed on day 14 after the last Laser Doppler study.

### Blood samples for determining plasma levels of microparticles

Blood samples (10 mL) were obtained at 9:00 am from study subjects for individual analysis according to the procedure and protocol outlined in our previous study [24]. In brief, peripheral blood was collected in acid citrate dextrose (ACD) vacutainer tubes. To prepare platelet-rich plasma, the peripheral blood (1.5 mL) was centrifuged at 2500 × g at 4°C for 15 min without acceleration or break. The 250 μL plasma samples were thawed and centrifuged for 10 min at 19,800 × g at 4°C, and then collected for investigation of MPs smaller than 1.0 μm. Size calibration was conducted with 1.0 μm beads (Invitrogen, Carlsbad, CA). All buffers were sterile-filtered with a 0.2 μm filter.

In the present study, the pellet and non-purification of the MPs were utilized. Additionally, because a large amount of MPs should be utilized for one animal, thus MPs were obtained from a pool of patients with the same pathology for one individual study.

### Measurement of blood flow with laser doppler (Figure 1)

**Figure 1:**
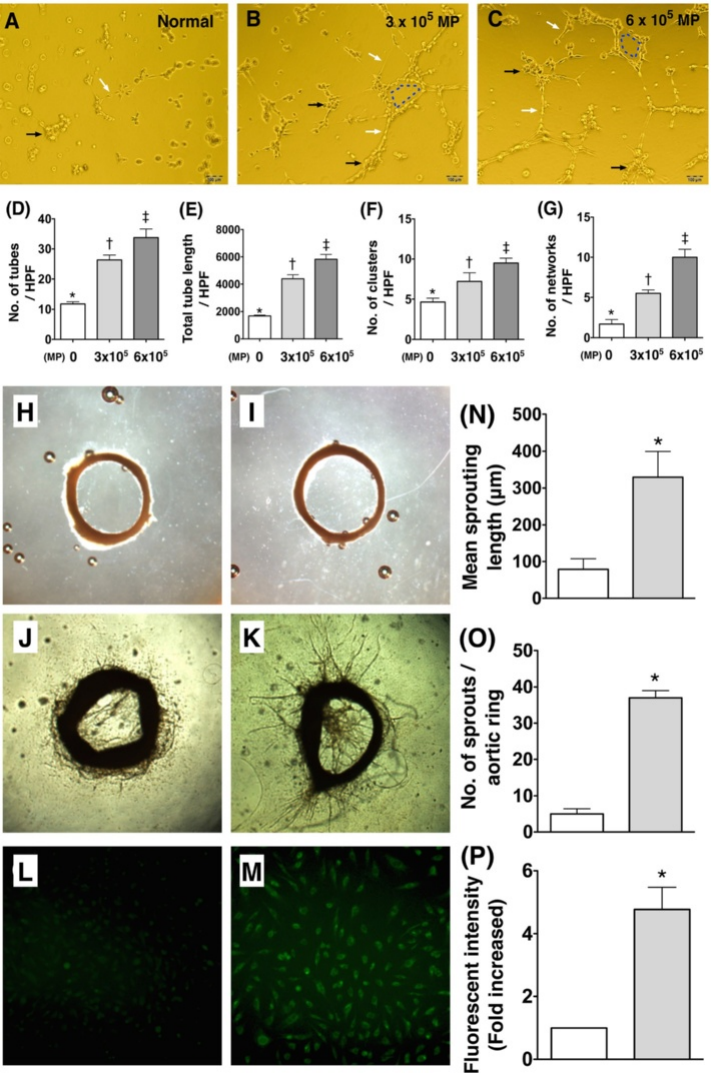
**Upper Panel**) **Matrigel Assay for angiogenesis with and without Patient**’**s Lung cancer-** **derived microparticles** **(Lc-** **MPs)** **treatment** **(n =** **6).** **A to** **C)** After 5-hour cell culture [1.0 × 10^4^ human umbilical vein endothelial cells (HUVECs)], Matrigel-assay angiogenesis was observed by microscopic findings (100×) in without **(A)** and with Lc-MPs treatment [3.0 × 10^5^ **(B)** and 6.0 × 10^5^ **(C)** MPs, respectively]. **D)** Analytical results of number of tubules (white arrows), * vs. other groups with different symbols (*, †, ‡), p < 0.001. **E)** Analytical results of total tubular length, * vs. other groups with different symbols (*, †, ‡), p < 0.001. **F)** Analytical results of cluster formation (black arrows), * vs. other groups with different symbols (*, †, ‡), p < 0.001. **G)** Analytical results of network formation (blue dot line), * vs. other groups with different symbols (*, †, ‡), p < 0.001. All statistical analyses were performed by one-way ANOVA, followed by Bonferroni multiple comparison post hoc test. Lower Panel) Aortic-ring (AR) angiogenesis and nitric oxide (NO) production after lung-cancer microparticles (Lc-MPs) treatment (n = 6). **H & I)** By day-5 cell culturing, the angiogenesis did not differ between with and without Lc-MPs treatment. **J & K)** By day-12 cell culturing, the AR angiogenesis was significantly enhanced in with **(K)** than in without **(J)** Lc-MP treatment. **N)** Mean sprouting length, p < 0.0001, * vs. control group; **O)** Number of sprouts around aortic ring, p < 0.0001, * vs. control group. **L & M)** Immunofluorescent microscopic finding of NO production by 6-hour after cell culture; **P)** The statistically analytic results of fluorescent intensity of NO expression, p < 0.0001, * vs. control group.

The procedure and protocol were based on our previous reports [25,26]. In brief, rats were anesthetized by inhalation of isoflurane (2.0%) prior to CLI induction and at days 2, 7, 14 after CLI induction prior to sacrifice. The rats were placed supine on a warming pad (37°C) and blood flow was detected in both inguinal areas by a Laser Doppler scanner (moorLDLS, Moor, Co. UK). The ratio of flow in the left (ischemic) leg and right (normal) leg was computed. By day 14, the rats were sacrificed and the quadriceps muscle was collected for individual study. Additionally, the lungs were collected to determine whether tumorigenesis was present or absent.

### Cell culture

Commercialized human umbilical vein endothelial cells (HUVECs) (BCRC) were utilized for the current study. Cells were first grown at 37°C in a 100 mm culture dish (Falcon) in M199 (Gibco) medium with endothelial cell growth supplement, heparin, and 20% fetal bovine serum (FBS) (Gibco). Cells of the third to fifth generation were used in the current study (Additional file 1).

### Method for determining Rat aortic-ring angiogenesis

Aortic-ring angiogenesis was conducted assay in twenty four-well tissue culture plates were embed with 150 μL of 1 mg/ml type I collagen (BD Biosciences, NJ) and allowed to gel for 60 minutes at 37°C and 5% CO_2_. The rat thoracic aorta was excised from 5- to 8-week-old Sprague Dawley rat, then remove all extraneous tissue and branching vessels with forceps and a scalpel. The aorta was cut into 1 mm of cross-section, placed on collagen-coated wells then filled with 500 μL of serum free MCDB131 medium. These rings were incubated for about 24 hours at 37°C and 5% CO_2_. After 24 hours, aorta rings were treated or non-treatment with micro-particles (6.0 x 10^5^ MPs) for 12 days and photographed at first day and 12 days with 12.5x magnification. The number and length of sprouting vessels were quantified by OLYMPUS DP72 software. Experiments were repeated two times.

### Western blot analyses

The procedure and protocol were based on our previous reports [25,26]. Equal amounts (50 μg) of protein extracts were loaded and separated by SDS-PAGE using acrylamide gradients. After electrophoresis, the separated proteins were transferred electrophoretically to a polyvinylidene difluoride (PVDF) membrane (Amersham Biosciences). Nonspecific sites were blocked by incubation of the membrane in blocking buffer [5% nonfat dry milk in T-TBS (TBS containing 0.05% Tween 20)] overnight. The membranes were incubated with the indicated primary antibodies [cytochrome c (Cyt c) (1: 2000, BD, mouse monoclonal), Bax (1: 1000, Abcam, rabbit polyclonal), caspase 3 (1:1000, Cell Signaling, rabbit monoclonal), poly(ADP-ribose) polymerase (PARP) (1:1000, Cell Signaling, rabbit polyclonal), Bcl-2 (1:200, Abcam, rabbit polyclonal), endothelial nitric oxide synthase (eNOS) (1: 1000, Abcam, rabbit polyclonal), VEGF (1:1000, Abcam, mouse monoclonal), SDF-1α (1:1000, Cell Signaling, rabbit polyclonal), CXCR4 (1:1000, Abcam, rabbit polyclonal), CD31 (1:3000, Abcam, mouse monoclonal), angiopoietin (1:1000, Millipore, rabbit polyclonal) hepatocyte growth factor (HGF) (1:1000, Abcam, Rabbit polyclonal), b-FGF (1:1000, Abcam, rabbit polyclonal), transforming growth factor (TGF)-β ( 1:500, Abcam, rabbit polyclonal), Smad3 (1:1000, Cell Signaling, rabbit polyclonal), bone morphogenetic protein (BMP)-2 (1:500, Abcam, rabbit polyclonal), Smad1/5 (1:1000, Cell Signaling, rabbit polyclonal), Actin (1:10000, Chemicon, mouse monoclonal)] for 1 hour at room temperature. Horseradish peroxidase-conjugated anti-rabbit or mouse IgG (1: 2000, Cell Signaling) was used as a secondary antibody for one hour at room temperature. The washing procedure was repeated eight times within one hour, and immunoreactive bands were visualized by enhanced chemiluminescence (ECL; Amersham Biosciences) and exposure to Biomax L film (Kodak). For purposes of quantification, ECL signals were digitized using Labwork software (UVP).

### Oxidative stress reaction in lower limb muscles

The Oxyblot Oxidized Protein Detection Kit was purchased from Chemicon (S7150). DNPH derivatization was carried out on 6 μg of protein for 15 minutes according to manufacturer’s instructions. One-dimensional electrophoresis was carried out on 12% SDS/polyacrylamide gel after DNPH derivatization. Proteins were transferred to nitrocellulose membranes which were then incubated in the primary antibody solution (anti-DNP 1: 150) for 2 h, followed by incubation with secondary antibody solution (1:300) for 1 hr at room temperature. The washing procedure was repeated eight times within 40 minutes. Immunoreactive bands were visualized by enhanced chemiluminescence (ECL; Amersham Biosciences) which was then exposed to Biomax L film (Kodak). For quantification, ECL signals were digitized using Labwork software (UVP). For oxyblot protein analysis, a standard control was loaded on each gel.

### Immunofluorescent (IF) measurement

The procedure and protocol were described in our previous studies [25,26]. For IF staining, cryo-sections were fixed with cold acetone for 3 minutes and then incubated with primary antibody specifically against CD31 (1:200, Serotec), vWF (1:100, Millipore), VEGF (1:100, Abcam), CXCR4 (1:100, Santa Cruz), SDF-1α (1:200, Santa Cruz) at 4°C overnight. After being washed with PBS, muscle sections were incubated with Alexa Fluor 594-conjugated goat anti-mouse IgG secondary antibodies for 30 minutes at room temperature, followed by counter-staining with DAPI. Fluorescent signals were observed with fluorescent-equipped microscope (IX-41, Olympus).

### Quantification of vessel density in limb ischemic area

The procedure and protocol were according to our previous reports [25,26]. Briefly, immunohistochemical (IHC) staining of blood vessels was performed with α-SMA (1:400) as primary antibody at room temperature for 1 h, followed by washing with PBS thrice. Ten minutes after the addition of anti-mouse-HRP conjugated secondary antibody, the tissue sections were washed with PBS thrice. Then 3,3’ diaminobenzidine (DAB) (0.7 gm/tablet) (Sigma) was added, followed by washing with PBS thrice after one minute. Finally, hematoxylin was added as a counter-stain for nuclei, followed by washing twice with PBS after one minute. Three sections of quadriceps were analyzed in each rat. For quantification, three randomly selected HPFs (200×) were analyzed in each section. The mean number per HPF for each animal was then determined by summation of all numbers divided by 9.

### Statistical analyses

Quantitative data are expressed as mean ± SD. Statistical analysis was performed by ANOVA followed by Bonferroni multiple-comparison *post hoc* test. All analyses were conducted using SAS statistical software for Windows version 8.2 (SAS institute, Cary, NC). A probability value <0.05 was considered statistically significant.

## Results

### Microparticles promoted angiogenesis, NO production, cell proliferation and VEGFR2 protein expression of HUVECs (Figures 1 and 2)

**Figure 2:**
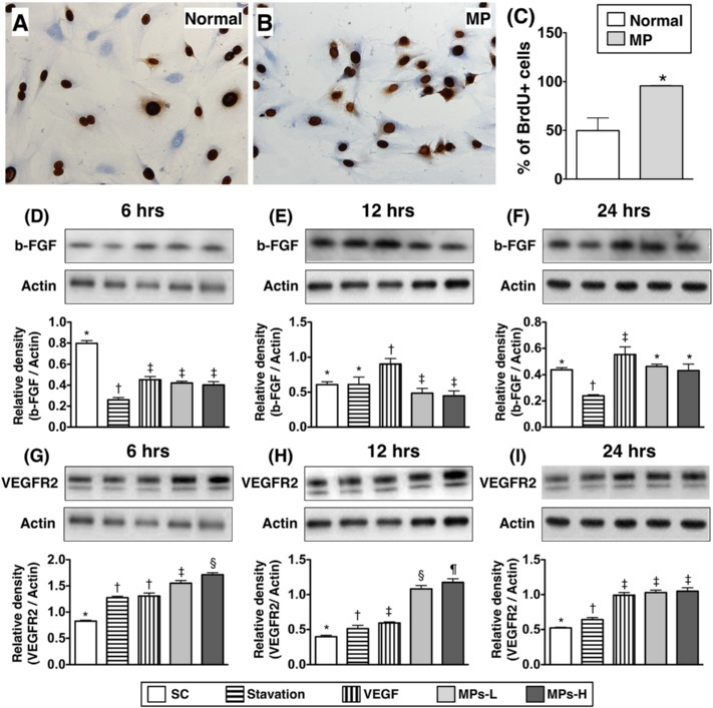
**HUVEC proliferation and protein expressions of pro-** **angiogenesis factors after lung-** **cancer microparticles** **(Lc**-**MPs)** **treatment** **(n =** **6).** **A & B)** Immunohistochemical stain of BrdU uptake by HUVECs (gray color). **C)** The statistically analytic results of BrdU+cells, p < 0.0001, * vs. control group. **D**, **E & F)** As compared with control group, the protein expression of basic-fibroblast growth factor (b-FGF) was not enhanced by Lc-MP treatment at the three time intervals (6, 12 and 24 h) of HUVEC culture. However, as compared with that of control group, the protein expression of b-FGF were significantly increased in HUVECs at time intervals of 12 and 24 h after vascular endothelial cell growth factor (VEGF) (50 ng/mL) treatment. * vs. other groups with different symbols, p < 0.001. **G,** **H & I)** As compared with control group, the protein expression of VEGFR2 in HUVECs were significantly augmented at 6 h and 12 h intervals and maintained at 24 h following Lc-MP treatment. * vs. other groups with different symbols, p < 0.001. Additionally, this protein expression in HUVECs showed an identical pattern of b-FGF at time intervals of 12 and 24 h after VEGF (50 ng/mL) treatment. All statistical analyses were performed by one-way ANOVA, followed by Bonferroni multiple comparison post hoc test. Symbols (*, †, ‡, §, ¶) indicate significance (at 0.05 level).

Figure 1 (Upper Panel) (A to G) shows the effect of Lc-MPs on angiogenesis at 5-hour HUVEC culture. As compared with control group, the cluster, tubular, and network formations on Matrigel assay were significantly increased after the two regimens of Lc-MP treatment (3.0 ×10^5^ and 6.0 × 10^5^) at 5-hour culturing interval. Intriguingly, these parameters were significantly increased in high-dose MP than that of low-dose MP treatment in this culturing time interval.

Figure 1 (Lower Panel H to P) shows the impact of Lc-MPs on angiogenesis of aortic ring. As expected, the angiogenesis of aortic ring was significantly increased with Lc-MP treatment compared with that without. Additionally, the impact of Lc-MP treatment on NO production in HUVECs was assayed in the present study. As expected, NO production was significantly enhanced in HUVECs after Lc-MP treatment compared with the non-treatment group.

Figure 2 (A to C) showed the effect of MP treatment on BrdU uptake by HUVECs, an indicator of cell proliferation. Consistent with the findings of NO production, BrdU uptake by HUVECs was also augmented by Lc-MP treatment, signifying an enhanced cellular proliferation after Lc-MP treatment.

As compared with control group, the Lc-MP treatment did not affect the protein expression of b-FGF in HUVECs at the three time intervals (i.e., at 6, 12 and 24 h) (Figure 2-D to F). This finding suggests that the MP-induced angiogenesis might not be through the b-FGF angiogenesis signaling pathway. However, the protein expression of this angiogenesis factor in HUVECs was significantly enhanced by VEGF treatment at 12 h and 24 h intervals.

The protein expression of VEGFR2 in HUVECs were significantly increased at 6 h and 12 h intervals and maintained at 24 h following Lc-MP treatment (Figure 2-G to I). These findings imply that VEGFR2 might be one of the crucial signaling pathways (i.e., interaction between ligand and receptor) for MPs to promote the capacity of angiogenesis among HUVECs.

### Laser doppler analysis of blood flow (Figure 3: upper panel)

**Figure 3:**
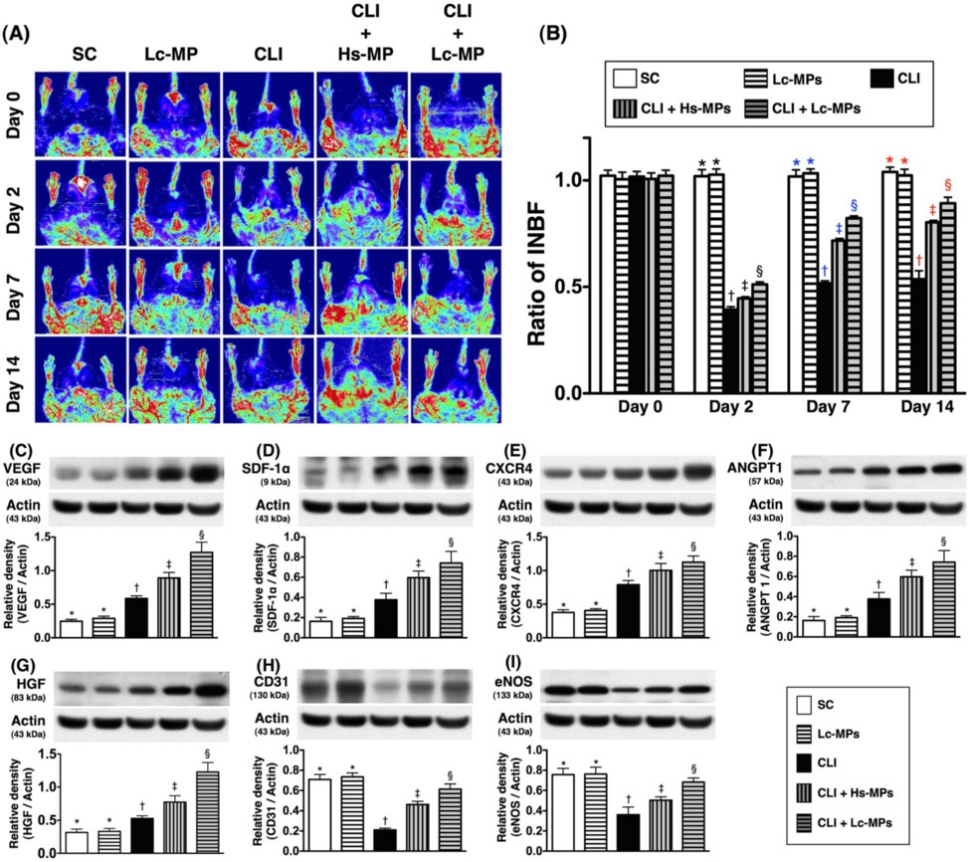
**Upper Panel)** **Ratio of ischemic/** **normal blood flow** **(INBF)** **by Laser Doppler scan on day 0 prior to procedure and on days 2,** **7 and 14 after critical limb ischemia** **(CLI)** **procedure** **(n =** **10).** **A)** Showing normal blood flow at baseline, days 2, 7 and 14 of both hind limbs among five groups. **B)** Analytical results of ratios of ischemic (left limb)/normal (right limb) blood flow (INBF) Laser Doppler at day 0 prior to CLI procedure) and days 2, 7 and 14 after CLI procedure. By day 2, * vs. other groups with different symbols (*, †, ‡, §), p < 0.0001; By day 7, * vs. other groups with different symbols (*, †, ‡, §), p < 0.0001; By day 14, * vs. other groups with different symbols (*, †, ‡, §), p < 0.0001. Lower Panel) Protein expressions of angiogenesis factors of ischemic quadriceps on day 14 following critical limb ischemia (CLI) procedure (n = 8). **C)** Protein expression of vascular endothelial growth factor (VEGF); **D)** Protein expression of stromal cell-derived factor (SDF)-1α; **E)** Protein expression of CXCR4; **F**) Protein expression of angiopoietin 1 (ANGPT 1); * vs. other groups with different symbols (*, †, ‡, §), p < 0.001. **G)** Protein expression of hepatocyte growth factor (HGF); **I)** Protein expression of CD31; Protein expression of endothelial nitric oxide synthase (eNOS); * vs. other groups with different symbols (*, †, ‡, §), p < 0.005. All statistical analyses were performed by one-way ANOVA, followed by Bonferroni multiple comparison post hoc test (n = 7). Symbols (*, †, ‡, §) indicate significance (at 0.05 level). SC = sham control; CLI = critical limb ischemia; Lc-MPs = lung-cancer patient-derived microparticles; Hs-MPs = Healthy subject-derived microparticles.

Figure 3 (Upper Panel) showed that the ratios of ischemic/normal blood flow (INBF) did not differ among the five groups, [i.e. sham control (SC) (group 1)], SC + Lc-MPs (group 2), CLI only (group 3), CLI + Hs-MPs (group 4), and CLI + Lc-MPs (group 5) on day 0 prior to the CLI procedure. There was also no significant difference between groups 1 and 2, but it was significantly and progressively reduced in groups 5 to 3 as compared with groups 1 and 2 on day 2 after CLI induction. By post-operative days 7 and 14, the ratio of INBF was significantly reduced in group 3 compared to that in other groups, significantly reduced in groups 4 and 5 than that in groups 1 and 2, and notably reduced in group 4 than that in group 5, but it did not differ between groups 1 and 2 (Figure 3). These findings imply that Lc-MP was more effective than Hs-MP in restoring blood flow in the ischemic limb.

### The protein expressions of Pro-angiogenic factors in ischemic quadriceps by Day 14 after CLI procedure (Figure 3: lower panel)

**Figure 4:**
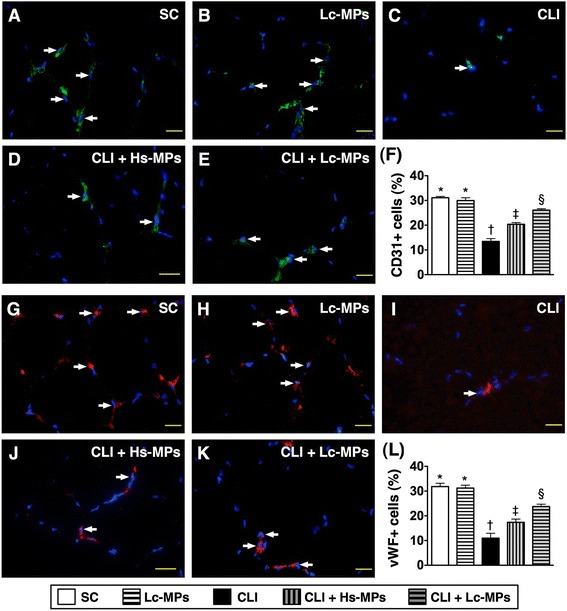
**Immunofluorescence** **(IF)** **microscope** **(400×)** **findings of von Willebrand factor** **(vWF)** ^**+**^ **and CD31** ^**+**^ **stained cells in ischemic quadriceps by day 14 following CLI procedure** **(n =** **8).** **A to E)** Showing the IF stain of CD31^+^ cells (white arrows) in five groups. **F)** * vs. other groups with different symbols (*, †, ‡, §), p < 0.0001. **G to K)** Illustrating the IF stain of vWF^+^ cells (white arrows) in five groups. **L)** * vs. other groups with different symbols (*, †, ‡, §), p < 0.0001. Scale bars in right lower corner represent 20 μm. All statistical analyses were performed by one-way ANOVA, followed by Bonferroni multiple comparison post hoc test. Symbols (*, †, ‡, §) indicate significance (at 0.05 level). SC = sham control; CLI = critical limb ischemia; Lc-MPs = lung-cancer patient-derived microparticles; Hs-MPs = Healthy subject-derived microparticles.

Figure 3 (Lower Panel) showed that the protein expressions of VEGF (Figure 3-C), SDF-1α (Figure 3-D), CXCR4 (Figure 3-E), angiopoietin-1 (Figure 3-F) and HGF (Figure 3-G) were lowest in groups 1 and 2 and highest in group 5, and significantly higher in group 4 than those in group 3, but they were similar between groups 1 and 2. These findings suggest that expressions of these angiogenesis markers, which only occur in condition of ischemic stress, were significantly up-regulated in response to Hs-MP and further notably reinforced after Lc-MP treatment. Additionally, the protein expression of CD31 (Figure 4-H) and eNOS (Figure 3-I), two indices of integrity of endothelial function and angiogenesis, were lowest in group 3 and highest in groups 1 and 2, and significantly higher in group 5 than in groups 4, but they exhibited no difference between groups 1 and 2.

### Immunofluorescent examination of Pro-angiogenic cells in ischemic quadriceps by Day 14 after CLI procedure (Figures 4, 5, and 6)

**Figure 5:**
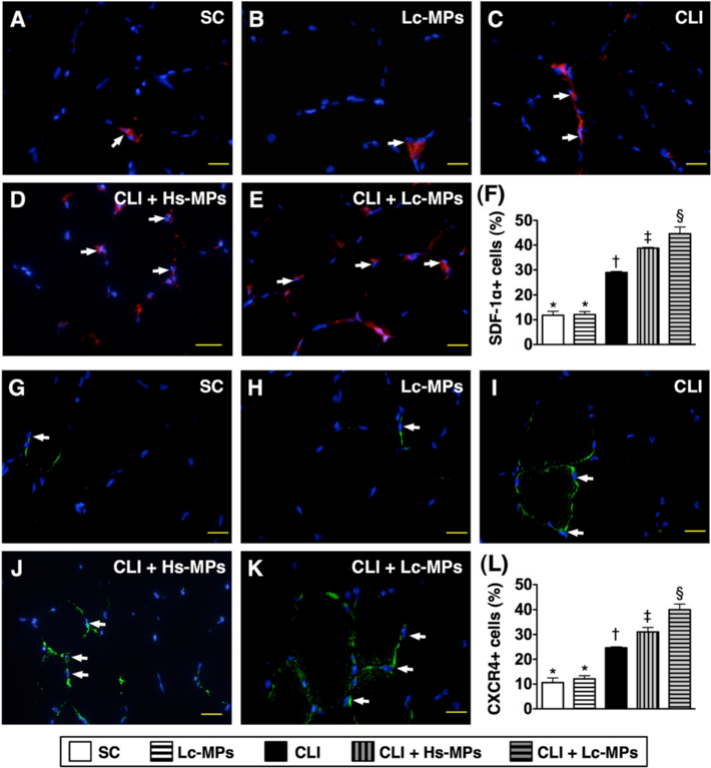
**Immunofluorescence** **(IF)** **microscope** **(400×)** **findings of stromal cell**-**derived factor** **(SDF)-** **1α** ^**+**^ **and CXCR4** ^**+**^ **stained cells in ischemic quadriceps by day 14 following CLI procedure** **(n =** **8).** **A to E)** Showing the IF stain of SDF-1α^+^ cells (white arrows) in five groups. **F)** * vs. other groups with different symbols (*, †, ‡, §), p < 0.0001. **G to K)** Illustrating the IF stain of CXCR4^+^ cells (white arrows) in five groups. **L)** * vs. other groups with different symbols (*, †, ‡, §), p < 0.0001. Scale bars in right lower corner represent 20 μm. All statistical analyses were performed by one-way ANOVA, followed by Bonferroni multiple comparison post hoc test. Symbols (*, †, ‡, §) indicate significance (at 0.05 level). SC = sham control; CLI = critical limb ischemia; Lc-MPs = lung-cancer patient-derived microparticles; Hs-MPs = Healthy subject-derived microparticles.

**Figure 6:**
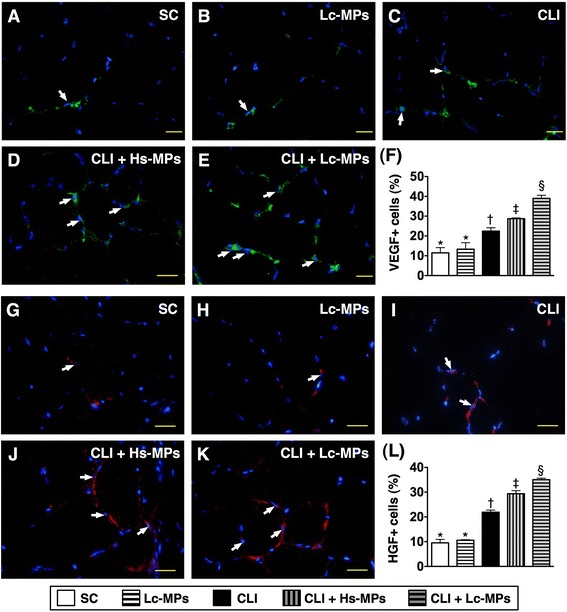
**Immunofluorescence** **(IF)** **microscope** **(400×)** **findings of vascular endothelial growth factor** **(VEGF)** ^**+**^ **and hepatocyte growth factor** **(HGF)** ^**+**^ **stained cells in ischemic quadriceps by day 14 following CLI procedure** **(n =** **8)** **. A to E)** Showing the IF stain of VEGF^+^ cells (white arrows) in five groups. **F)** * vs. other groups with different symbols (*, †, ‡, §), p < 0.0001. **G to K)** Illustrating the IF stain of HGF^+^ cells (white arrows) in five groups. **L)** * vs. other groups with different symbols (*, †, ‡, §), p < 0.0001. Scale bars in right lower corner represent 20 μm. All statistical analyses were performed by one-way ANOVA, followed by Bonferroni multiple comparison post hoc test. Symbols (*, †, ‡, §) indicate significance (at 0.05 level). SC = sham control; CLI = critical limb ischemia; Lc-MPs = lung-cancer patient-derived microparticles; Hs-MPs = Healthy subject-derived microparticles.

The results of IF staining showed that the numbers of CD31^+^ (Figure 4-A to F) and vWF^+^ (Figure 4-G to L) cells, two endothelial cell markers, were significantly lower in group 3 than in other groups, significantly lower in groups 4 and 5 than in groups 1 and 2, and significantly lower in group 4 than in group 5, but they revealed no difference between groups 1 and 2. Moreover, the numbers of SDF-1α^+^ (Figure 5 A to F), CXCR4^+^ (Figure 5 G to L), VEGF^+^ (Figure 6 A to F) and HGF^+^ (Figure 6-G to L) cells, four indicators of angiogenesis biomarkers, were highest in group 5 and lowest in groups 1 and 2, and significantly higher in group 4 than in group 3, but they exhibited no difference between groups 1 and 2. These findings imply that Lc-MP had higher capacity than Hs-MP of promoting the retention of pro-angiogenic cells in the ischemic limbs.

### Quantitative analysis of IHC staining of ischemic quadriceps and hematoxylin-eosin staining of lung tissue on Day 14 after CLI procedure (Figure 7)

**Figure 7:**
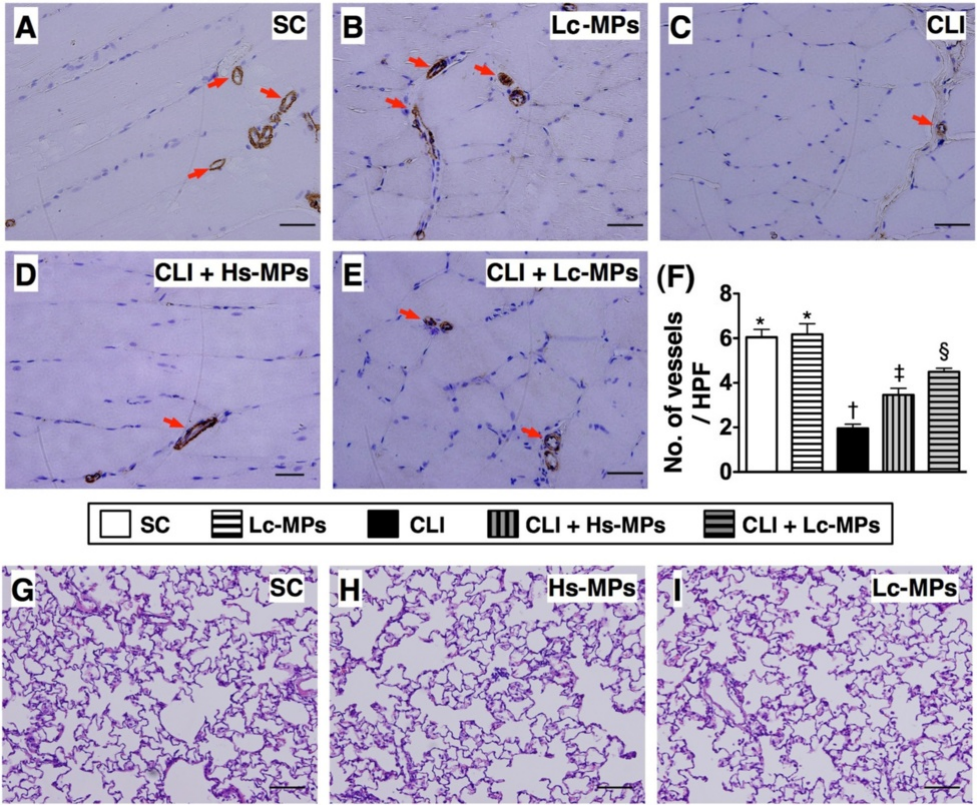
**Immunohistochemical** **(IHC) (** **200×)** **staining of alpha-** **smooth muscle actin for quantification of small vessel** **(≤25.0 μm)** **in ischemic quadriceps at day 14 after CLI induction** **(n =** **8** **).** **A to E)** Indicating IHC stain of number of small vessels (red arrows) in five groups. **F)** * vs. other groups with different symbols (*, †, ‡, §), p < 0.0001. Scale bars in right lower corner represent 50 μm. **G to I)** Demonstrating the H.E. staining for the lung parenchyma in SC + Lc-MPs **(G)**, CLI + Hs-MPs **(H)**, and CLI + Lc-MPs. No tumorigenesis was found in these three groups of animals. All statistical analyses were performed by one-way ANOVA, followed by Bonferroni multiple comparison post hoc test. Symbols (*, †, ‡, §) indicate significance (at 0.05 level). SC = sham control; CLI = critical limb ischemia; Lc-MPs = lung-cancer patient-derived microparticles; Hs-MPs = Healthy subject-derived microparticles.

The results of IHC staining showed remarkably lower number of small vessels (defined as < 15 μm) in group 3 than in other groups (Figure 7-A to F). It was also notably lower in groups 4 and 5 than that in groups 1 and 2, and significantly lower in group 4 than that in group 5, but it did not differ between groups 1 and 2. These findings once more suggest that augmented angiogenesis/neovascularization only develop in situation of ischemic stimulation. Moreover, it was notably strengthened in the Lc-MPs treatment group compared with animals after Hs-MPS treatment in response to ischemic stress. Importantly, no sign of tumorigenesis was noted in the ischemic limbs or in lung tissue (Figure 7-G to I).

### Protein expressions of fibrosis, anti-fibrosis, apoptosis, and anti-apoptosis biomarkers in ischemic quadriceps by Day 14 after CLI procedure (Figures 8 and 9)

**Figure 8:**
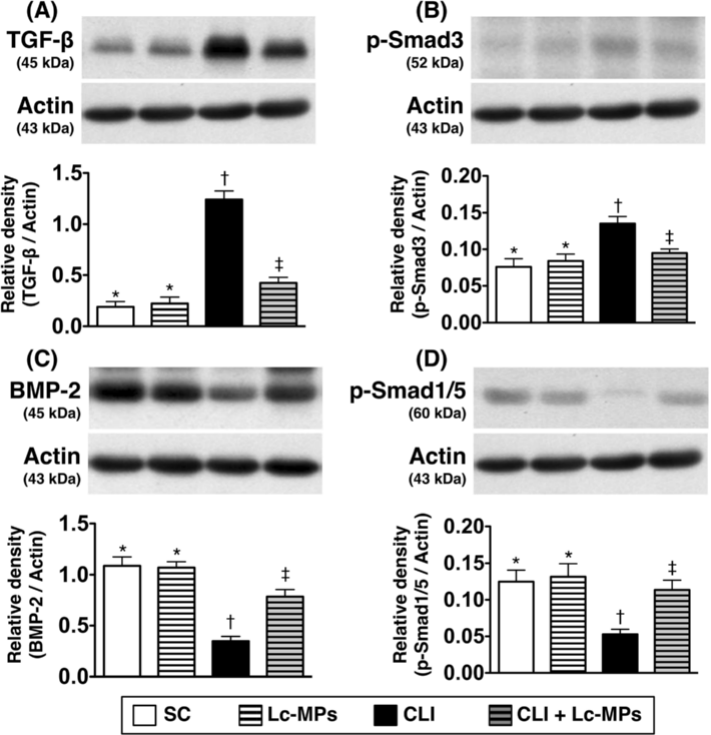
**Protein expressions of fibrotic and anti**-**fibrotic biomarkers in ischemic quadriceps on day 14 following critical limb ischemia** **(CLI)** **procedure** **(n =** **8).** **A)** Protein expression of transforming growth factor (TGF)-β in four groups (SC = sham control; SC + Lc-MPs = sham control + lung-cancer patient-derived microparticles; CLI = critical limb ischemia; CLI + Lc-MPs = critical limb ischemia + lung-cancer patient-derived microparticles), * vs. other groups with different symbols (*, †, ‡), p < 0.001. **B**) Protein expression of phosphorylated (p)-Smad3 in four groups, * vs. other groups with different symbols (*, †, ‡), p < 0.01. **C)** Protein expression of bone morphogenetic protein (BMP)-2 in four groups, * vs. other groups with different symbols (*, †, ‡), p < 0.001. **D)** Protein expression of p-Smad1/5 in four groups, * vs. other groups with different symbols (*, †, ‡), p < 0.01. All statistical analyses were performed by one-way ANOVA, followed by Bonferroni multiple comparison post hoc test. Symbols (*, †, ‡) indicate significance (at 0.05 level).

**Figure 9:**
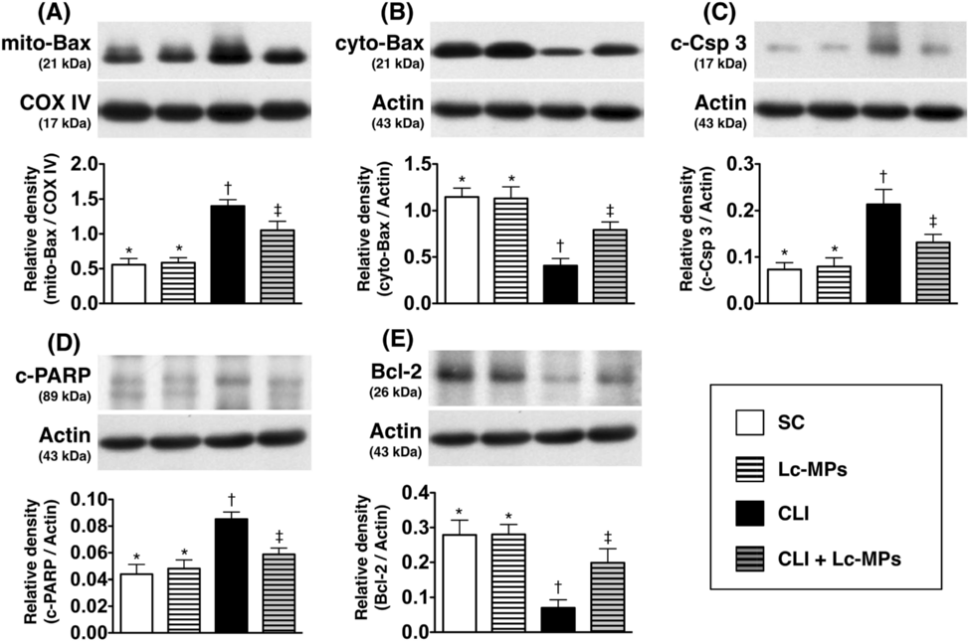
**Protein expressions of apoptotic and anti**-**apoptotic biomarkers in ischemic quadriceps on day 14 following critical limb ischemia** **(CLI)** **procedure** **(n =** **8).** **A)** Protein expression of mitochondrial Bax (mito-Bax) in four groups (SC = sham control; SC + Lc-MPs = sham control + lung-cancer patient-derived microparticles; CLI = critical limb ischemia; CLI + Lc-MPs = critical limb ischemia + lung-cancer patient-derived microparticles), * vs. other groups with different symbols (*, †, ‡), p < 0.001. **B)** The protein expression of cytosolic Bax (cyto-Bax) in four groups, * vs. other groups with different symbols (*, †, ‡), p < 0.001. **C)** Protein expression of cleaved caspase (c-Csp) 3 in four groups, * vs. other groups with different symbols (*, †, ‡), p < 0.001. **D)** Protein expression of cleaved poly (ADP-ribose) polymerase (PARP), * vs. other groups with different symbols (*, †, ‡), p < 0.001. **E)** The protein expression of Bcl-2, * vs. other groups with different symbols (*, †, ‡), p < 0.001.

The protein expressions of TGF-β (Figure 8-A) and p-Smad3 (Figure 8-B), two fibrotic biomarkers, were significantly increased in CLI-Lc-MPs and further increased in CLI than in SC and SC-Lc-MPs, but it displayed no differences between later two groups. On the other hand, the protein expressions of BMP-2 (Figure 8-C) and p-Smad1/5 (Figure 8-D), two indices of anti-fibrosis, showed an opposite pattern of fibrotic biomarkers among the four groups. Additionally, the protein expression of mitochondrial Bax (Figure 9-A), cytosolic Bax (Figure 9-B) cleaved (i.e., active form) caspase 3 (Figure 9-C), and cleaved PARP (Figure 9-D), and cytosolic Bax (a versed pattern of mitochondrial Bax) (Figure 9-B), the indicators of apoptosis, showed an identical pattern compared to that of fibrosis, whereas the protein expressions of Bcl-2 (Figure 9-E), an anti-apoptotic marker, showed a reversed pattern compared to that of fibrosis among the four groups.

## Discussion

This study, which investigated the impact of MPs on promoting angiogenesis and restoration of blood flow in ischemic limbs in a rodent model, yielded several striking implications. First, the results of HUVEC culturing study proved that Lc-MP treatment enhanced angiogenesis, cell proliferation, and NO production as well as VEGFR2 protein expressions, suggesting that MP treatment augmented angiogenesis mainly through eliciting the VEGF signaling pathway. Second, both ex vivo (i.e., aortic ring) and in vivo (i.e., ischemic limb) studies demonstrated that MP therapy enhanced angiogenesis. Third, the remarkably increased blood flow to the ischemic limbs after MP treatment suggests that MPs may also play a principal role in restoring the microcirculation in other ischemia-induced organ injuries. Fourth, lung cancer-derived MPs were more effective than those derived from healthy subjects in enhancing angiogenesis and restoring blood flow in the ischemic regions. Importantly, our in vitro and in vivo studies provided an outcome of scientifically mutual authentication.

One important finding of the in vitro study using aortic-ring and HUVEC cultures is the angiogenesis-promoting capacity of Lc-MPs. In particular, Lc-MPs were shown to augment HUVEC proliferation and NO production (i.e., an indicator of angiogenesis). Our findings are consistent with those of previous studies that demonstrated MP-induced angiogenesis and differentiation of bone marrow-derived endothelial progenitor cells [27-29]. Another important finding of the present study is the in vitro demonstration of markedly up-regulated VEGFR2 protein expressions in HUVECs after Lc-MP treatment. In contrast, the protein expression of bFGF (i.e., angiogenesis molecules) in HUVECs did not show significant change in with and without Lc-PM treatment. Intriguingly, one recent study has suggested that MPs can act directly through the ligand/receptor interaction or indirectly on angiogenesis by modulating soluble factor production involved in endothelial cell differentiation, proliferation, migration, and adhesion [30]. Previous studies have suggested that the PPAR alpha and VEGF family of angiogenic factors and their receptors are essential for MP-induced differentiation and angiogenesis [29,31]. Additionally, another previous study has shown that circulating levels of PMs and circulating endothelial cells correlated with prognosis, and could be useful as prognostic markers in patients with advanced non-small cell lung cancer [32]. In this way, our finding is comparable to those of previous studies [29-32], suggesting that VEGF receptor may be one of the important signaling pathways in angiogenesis and cell differentiation. Target therapy is one of the common management strategies for advanced NSCLC. Recent data has suggested that anti-VEGF monoclonal antibody such as bevacizumab is an acceptable palliative drug for advanced NSCLC [5]. Accordingly, our data strengthen the support for potential clinical application of anti-VEGF regimen in this setting.

The most important finding in the present study is that, as compared to the SC group, administration of Lc-MPs to SC animals did not result in any significant change of blood flow in the ischemic limbs. On the other hand, while Hs-MP therapy significantly increased blood flow in the ischemic regions, Lc-MP further significantly enhanced blood flow in the CLI area. The finding, which was first identified by the present study, highlights the superior potency of Lc-MP to that of Hs-MP in enhancing angiogenesis which may help in explaining tumor sprouting and augmented angiogenesis for supporting tumor growth and metastasis. Our findings, therefore, support the finding of the previous study that MPs plays an essential role on the propagation of non-small cell lung cancer [32].

An essential finding in the current study is that, as compared to animals with ischemic limbs without treatment, Hs-MP substantially enhanced and Lc-MP further boosted the numbers of angiogenesis cells in the ischemic limbs. Another interesting finding in the present study is that the number of small vessels showed an identical pattern of changes compared to that of expression of the angiogenesis cells among these animals. Our findings also reinforce those of previous studies demonstrating enhancement of vasculogenesis after treatment with platelet-derived or ischemic muscle-derived MPs in experimental models of vascular injuries [33,34]. Another finding not previously reported is the lack of difference in the number of small vessels and pro-angiogenic cells in the healthy limbs between control animals with and without MP treatment. Again, these findings highlight the therapeutic role of MPs only under the condition of ischemia.

A principal finding in the present study is that the protein expressions of pro-angiogenic factors were markedly increased in CLI animals with MP treatment as compared to those without. Besides, the expressions of anti-apoptotic and anti-fibrotic biomarkers were remarkably higher, whereas the apoptotic and fibrotic biomarkers were notably lower in CLI animals with Lc-MP treatment than in those without. Our previous studies have also shown identical pattern of expressions of these biomarkers in CLI animals without treatment [25,26]. Another interesting finding is that there was no alternation of these biomarkers between control animals with and without MP treatment. Accordingly, our findings, in addition to being consistent with the results of our previous studies [25,26], once more explain the restoration of blood flow after MP treatment in a rodent model of CLI.

### Study limitations

**Figure 10:**
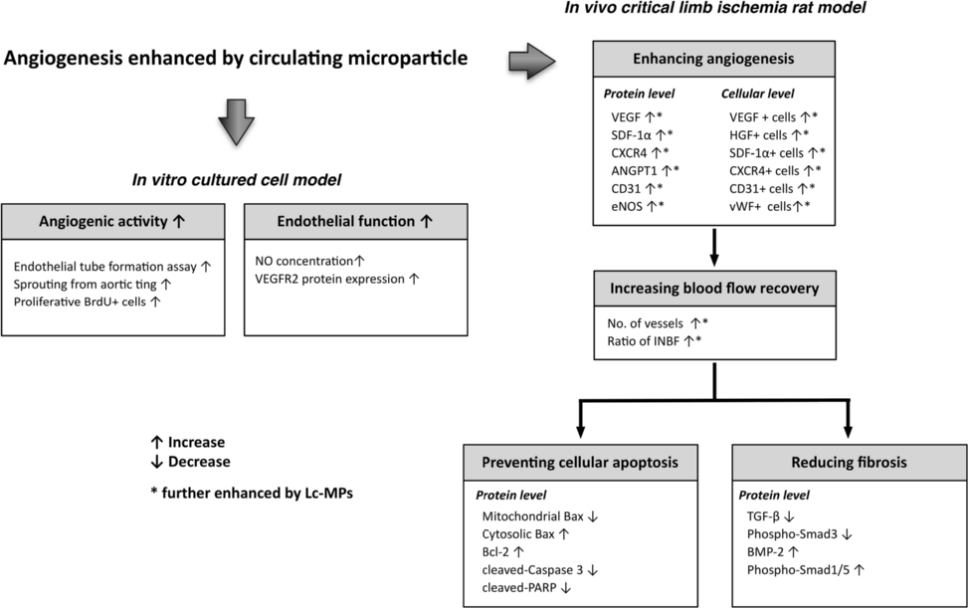
**Proposed mechanisms underlying the positive therapeutic effects of circulating microparticles on critical limb ischemia.** Lc-MPs = lung cancer-derived circulating microparticles; NO = nitric oxide; VEGFR2 = vascular endothelial growth factor receptor 2; SDF-1α = stromal cell-derived factor 1 alpha; ANGPT = angiopoietin; eNOS = endothelial nitric oxide synthase; HGF = hepatocyte growth factor; vWF = von Willebrand factor; IFBF = Ratio of ischemic to normal blood flow; PARP = poly (ADP-ribose) polymerase; TGF-β = transforming growth factor beta; BMP = bone morphogenesis protein.

This study has limitations. First, the potential long-term side-effect of tumorigenesis from lung cancer-derived MPs was not assessed in the current study. Second, since the present in vitro study (Figure 2) did not screen for all pro-angiogenic molecules/receptors acted on by Lc-MPs other than VEGFR2 and bFGF, no such information was available. Additionally, the findings from Figure 2 did not allow drawing the conclusion that effects of MPs from healthy subjects were inferior to those from cancer patients. Third, this study focused on the investigation of angiogenesis rather than on the tumor sprouting and metastasis. Fourth, we did not provide the effects of MPs from healthy subjects in Figures 8 and 9. Therefore, we did not know how specificity of the effects of Lc-MPs on these biomarkers. Finally, although extensive investigation has been performed in the current study, the exact mechanisms underlying the therapeutic effects of circulating MPs on improving the CLI are still unclear. The proposed mechanisms by which MP treatment salvaged CLI and improved the blood flow in ischemic area in a rodent CLI model have been summarized in Figure 10.

In conclusion, our data not only provide evidence that Lc-MP is more effective than Hs-MP in enhancing angiogenesis, vascularization, and restoration of blood flow in an animal model of CLI, but also suggest that Lc-MPs may play a key role in the propagation of lung cancer.

## Additional file

Additional file 1:
**Determination of the effects of Lc-MPs on Angiogenesis, Proliferation, Nitric Oxide (NO) Production, and Expressions of Angiogenesis Factors.** The procedures for determining angiogenesis were based on our previous report [35]. In brief, HUVECs (10^4^ cells) were incubated with MPs (3.0 x 10^5^ or 6.0 x 10^5^ particles); or vehicle for 72 hours. HUVECs were then plated on 96-well plates at 10^4^ cells/well in serum-free M199 culture medium mixed with cold Matrigel (Chemicon international) for 5 hour incubation at 37°C in 5% CO_2_, respectively. Three random images were taken for counting cluster, tube, and network formations. To investigate the effect of Lc-MPs on NO production, examination of NO production in HUVECs were performed by adding 5 µM 4-amino-5-methylamino-2’,7’-difluorofluorescein diacetate (DAF-FM Diacetate; Molecular Probes) at 37°C for 30 minutes. Cells were washed with PBS twice, and counterstained with Hoechst 33258 (0.5 µg/mL, Sigma) for 30 minutes in room temperature. To determine the impact of Lc-MPs on cellular proliferation, 5-bromodeoxyuridine (BrdU) (0.01 mM) was used. Following 48 hours of BrdU incubation, in situ detection was performed using the BrdU In-Situ Detection Kit (BD Biosciences Pharmingen, USA). To determine the impact of MPs on the protein expressions of VEGFR2 and basic-fibroblast growth factor (b-FGF), two indicators of angiogenesis, HUVECs (5.0×10^5^ cells) were cultured with low-dose (1.0 x 10^4^ particles) and high-dose (5.0 x 10^4^ particles) MPs for time courses of 6, 12 and 24 hrs (i.e., Cells were harvested after 6, 12, and 24hrs). Treatment of the HUVECs with the VEGF (50 ng/mL) served as a positive control. The cells then were collected for Western blot analysis.To assess the direct effect of Lc-MPs on angiogenesis in blood vessel, aortic ring (from rat ascending aorta) was cultured with Lc-MPs (i.e., ex vivo test) in M199 culture.
