# Supplementary material for: Administered circulating microparticles derived from lung cancer patients markedly improved angiogenesis, blood flow and ischemic recovery in rat critical limb ischemia
Source: J Transl Med. 2015 Feb 15;13:59. doi: 10.1186/s12967-015-0381-8 (PMC4369091; doi:10.1186/s12967-015-0381-8)
Supplement: Additional file 1: — Determination of the effects of Lc-MPs on Angiogenesis, Proliferation, Nitric Oxide (NO) Production, and Expressions of Angiogenesis Factors. The procedures for determining angiogenesis were based on our previous report [35]. In brief, HUVECs (104 cells) were incubated with MPs (3.0 x 10^5^ or 6.0 x 10^5^ particles); or vehicle for 72 hours. HUVECs were then plated on 96-well plates at 104 cells/well in serum-free M199 culture medium mixed with cold Matrigel (Chemicon international) for 5 hour incubation at 37°C in 5% CO2, respectively. Three random images were taken for counting cluster, tube, and network formations. To investigate the effect of Lc-MPs on NO production, examination of NO production in HUVECs were performed by adding 5 µM 4-amino-5-methylamino-2’,7’-difluorofluorescein diacetate (DAF-FM Diacetate; Molecular Probes) at 37°C for 30 minutes. Cells were washed with PBS twice, and counterstained with Hoechst 33258 (0.5 µg/mL, Sigma) for 30 minutes in room temperature. To determine the impact of Lc-MPs on cellular proliferation, 5-bromodeoxyuridine (BrdU) (0.01 mM) was used. Following 48 hours of BrdU incubation, in situ detection was performed using the BrdU In-Situ Detection Kit (BD Biosciences Pharmingen, USA). To determine the impact of MPs on the protein expressions of VEGFR2 and basic-fibroblast growth factor (b-FGF), two indicators of angiogenesis, HUVECs (5.0×10^5^ cells) were cultured with low-dose (1.0 x 10^4^ particles) and high-dose (5.0 x 10^4^ particles) MPs for time courses of 6, 12 and 24 hrs (i.e., Cells were harvested after 6, 12, and 24hrs). Treatment of the HUVECs with the VEGF (50 ng/mL) served as a positive control. The cells then were collected for Western blot analysis.To assess the direct effect of Lc-MPs on angiogenesis in blood vessel, aortic ring (from rat ascending aorta) was cultured with Lc-MPs (i.e., ex vivo test) in M199 culture. [file 12967_2015_381_MOESM1_ESM.docx]

**Supplemental method**

**Determination of the Effects of Lc-MPs on Angiogenesis, Proliferation, Nitric Oxide (NO) Production, and Expressions of Angiogenesis Factors**

The procedure and protocol for determining angiogenesis were based on our previous report [[27](#_ENREF_27)]. In brief, to evaluate the impact of Lc-MPs on angiogenesis, HUVECs (1.0 x 10^4^ cells) (n=6 per group) were incubated with MPs (3.0 x 10^5^ or 6.0 x 10^5^ particles); or vehicle (i.e., M199 culture medium) for 72 hours. The HUVECs were then plated on 96-well plates at 1.0×10^4^ cells/well in 150 µL serum-free M199 culture medium mixed with 50 µL cold Matrigel (Chemicon international) for 5 hour incubation at 37 °C in 5% CO_2_, respectively. Three random microscopic images (100x) were taken at each well for counting cluster, tube, and network formations with the mean values obtained.

To investigate the effect of Lc-MPs on NO production, HUVECs (2.0×10^5^ cells) were co-cultured with Lc-MPs (5.0 x 10^5^ particles) or vehicle (0.1% DMSO or PBS) at 37 °C for 6 hours. The HUVECs were then plated on glass coverslips and grown to sub-confluence for determination of fluorescence intensity from intracellular NO production by adding 5 µM of specific cell-permeable fluorescent probe 4-amino-5-methylamino-2’,7’-difluorofluorescein diacetate (DAF-FM Diacetate; Molecular Probes) to the cells which were cultured in serum-free medium and incubated at 37 °C for 30 minutes. DAF-FM, produced by cytosolic esterases, was converted in the presence of NO into Benzotriazole derivative (Excitation 492 nm; Emission 515 nm). Cells were washed with PBS twice, and counterstained with Hoechst 33258 (0.5 μg/mL, Sigma) for 30 minutes in room temperature. The samples were analyzed using fluorescence microscopy (Olympus Bx51).

To determine the impact of Lc-MPs on cellular proliferation, HUVECs (1.0×10^5^ cells) were co-cultured with MPs (6.0 x 10^5^ particles) or vehicle (M199 culture medium) for 72 hours. After 24 hours of culturing, 5-bromodeoxyuridine (BrdU) solution was added to attain a final concentration of 0.01 mM. Following 48 hours of BrdU incubation, in situ detection was performed using the BrdU In-Situ Detection Kit (BD Biosciences Pharmingen, USA) according to the manufacturer’s instructions. The percentage of proliferating cells was evaluated by counting BrdU-positive cells (brown) per 300 hematoxylin-stained nuclei at ×200 magnification. Three hundred nuclei were analyzed. The samples were analyzed using fluorescence microscopy (Olympus Bx51).

To determine the impact of MPs on the protein expressions of VEGF and basic-fibroblast growth factor (b-FGF), two indicators of angiogenesis, HUVECs (5.0×10^5^ cells) were cultured with low-dose (1.0 x 10^4^ particles) and high-dose (5.0 x 10^4^ particles) MPs for time courses of 6, 12 and 24 hrs (i.e., Cells were harvested after 6, 12, and 24hrs). Treatment of the HUVECs with the VEGF (50 ng/mL) served as a positive control. The cells then were collected for Western blot analysis.

**Determination of the Effects of Lc-MPs on Ex Vivo Angiogenesis**

To assess the direct effect of Lc-MPs on angiogenesis in blood vessel, aortic ring (from rat ascending aorta) was cultured with Lc-MPs (i.e., ex vivo test) in M199 culture medium.
